# A Novel Use of Allopurinol as A Quorum-Sensing Inhibitor in *Pseudomonas aeruginosa*

**DOI:** 10.3390/antibiotics10111385

**Published:** 2021-11-12

**Authors:** Ahmed Al Saqr, Mohammed F. Aldawsari, El-Sayed Khafagy, Moataz A. Shaldam, Wael A. H. Hegazy, Hisham A. Abbas

**Affiliations:** 1 Department of Pharmaceutics, College of Pharmacy, Prince Sattam Bin Abdulaziz University, Al-kharj 11942, Saudi Arabia; a.alsaqr@psau.edu.sa (A.A.S.); moh.aldawsari@psau.edu.sa (M.F.A.); 2 Department of Pharmaceutics and Industrial Pharmacy, Faculty of Pharmacy, Suez Canal University, Ismailia 41522, Egypt; 3 Department of Pharmaceutical Chemistry, Faculty of Pharmacy, Kafrelsheikh University, Kafr El-Sheikh 33511, Egypt; dr_moutaz_986@yahoo.com; 4 Department of Microbiology and Immunology, Faculty of Pharmacy, Zagazig University, Zagazig 44519, Egypt; waelmhegazy@daad-alumni.de (W.A.H.H.); hishamabbas2008@gmail.com (H.A.A.)

**Keywords:** quorum sensing, allopurinol, virulence inhibition, *Pseudomonas aeruginosa*

## Abstract

*Pseudomonas aeruginosa* can cause a variety of healthcare-associated infections by its arsenal of virulence factors. Virulence factor production is largely controlled by the cell-to-cell communication system termed quorum sensing (QS). Targeting QS may be a good approach to inhibit the production of virulence factors and attenuate pathogenicity without exerting selective stress on bacterial growth. This will greatly reduce the emergence of resistant mutants. In this work, we investigated the anti-virulence and anti-QS activities of the FDA-approved drug allopurinol against the *P. aeruginosa* PAO1 strain. Allopurinol at 200 µg/mL (1/10 MIC) significantly decreased the production of the QS-controlled *Chromobacterium violaceum* CV026 violet pigment violacein and other *P. aeruginosa* QS-controlled virulence factors phenotypically. Furthermore, allopurinol reduced the infiltration of *P. aeruginosa* and leucocytes and diminished the congestion in the liver and kidney tissues of infected mice. In silico study showed that allopurinol could compete with the autoinducers on binding to the receptors LasR and RhlR by hydrogen bonding. On the molecular level, qRT-PCR proved that allopurinol showed a significant downregulating effect on all tested QS-encoding genes that regulate virulence factor production. In summary, allopurinol is a promising QS inhibitor that may be useful in the future treatment of *P. aeruginosa* infection.

## 1. Introduction

The Gram-negative bacterium, *Pseudomonas aeruginosa*, is responsible for many types of infections such as those of the eye [1] and burn wounds [2] in addition to acute and chronic respiratory tract infections [3]. To cause such infection, *P. aeruginosa* employs its arsenal that includes a wide variety of virulence factors such as motility, various proteases, and toxins as reviewed [4,5,6,7]. *P. aeruginosa* is considered among the most major global health concerns due to the elevated levels of resistance to disinfectants and antibiotics [5,6]. In 2013, according to the Centers for Disease Control and Prevention (CDC), multidrug-resistant (MDR) *P. aeruginosa* strains accounted for approximately 13% of all *P. aeruginosa* infections and were the cause of death for over 400 individuals in the USA. During infection, *P. aeruginosa* habitually forms biofilms that confer a dramatic increase in resistance to antibiotics, even if it is susceptible when grown planktonically [5,8]. It is worth mentioning that the *P. aeruginosa* intrinsic resistance is owed basically to low membrane permeability in addition to diverse multidrug efflux pumps, mainly the MexXY–OprM and MexAB–OprM systems [9,10].

Quorum-sensing (QS) systems are common in bacterial pathogens, including *P. aeruginosa* [11,12,13], that employ them to regulate bacterial virulence. *P. aeruginosa* virulence is controlled by three QS systems, two of which are LuxI/LuxR types and one which is a non-LuxI/LuxR type, namely, the *Pseudomonas* quinolone signal (PQS) system [7]. *P. aeruginosa* QS systems have a hierarchical architecture controlled by the LasR/LasI system, which is responsible for the production and sensing of the signaling molecule *N*-3-oxo-dodecanoyl-L-homoserine lactone. This system activates the production of multiple virulence factors such as pyocyanin, protease, and elastase. Furthermore, LasR/LasI system activates the RhlR/RhlI QS system that controls the secretion and sensing of the autoinducer *N*-butyryl-L-homoserine lactone. This system is involved in the control of rhamnolipids and pyocyanin [14,15]. A third QS system in *P. aeruginosa* is a quinolone-dependent system termed the *Pseudomonas* quinolone system (PQS), which is regulated by LasR and RhlR [6,16]. As a result, QS inhibition could be useful for attenuating virulence, providing an alternative approach in addition to traditional antibiotic treatments [17,18,19,20,21].

The bacterial resistance to antibiotics is a worrisome problem that must be overcome to guarantee an effective antimicrobial treatment, especially in aggressive infections such as those observed in *P. aeruginosa* infections. There are several approaches that have been introduced to conquer bacterial resistance [5,17,20,21,22]. Targeting bacterial virulence is a promising strategy that provides a chance for the immune system to eradicate the bacterial invaders. Bearing in mind that QS is the virulence regulator that controls bacterial pathogenesis, it is logical to target QS to diminish bacterial pathogenesis. Bacterial QS has been targeted or quenched by a wide diversity of chemical moieties, giving special consideration to safe and known prescribed drugs [5,18,23]. The interest in repurposing approved drugs to target bacterial QS and virulence has increased over the last few years. In this context, we screened the anti-QS activities of several approved drugs [6,19,20,24,25,26]. The non-purine “allopurinol” is a xanthine oxidoreductase selective inhibitor that is clinically employed in the treatment of gout, as it diminishes the urate concentration [27,28]. Allopurinol has been used as eye drops (0.4%) to treat corneal burns [29], and it is a purine analog and a structural isomer of hypoxanthine that is a natural body purine [30]. Caffeine is a xanthine compound that shares with allopurinol its purine moiety [31]. Caffeine is reported to have anti-virulence and anti-QS activities against the *P. aeruginosa* PAO1 strain, and it has shown inhibiting activity against acyl-homoserine production and swarming motility in *P. aeruginosa* [32]. Such activity encouraged us to explore the anti-QS activities of allopurinol. This study aimed to investigate the possible allopurinol anti-QS activity and its effect on *P. aeruginosa* virulence factors to evaluate the possibility of its clinical application, for instance, topically in the treatment of *P. aeruginosa* eye infections.

## 2. Results

### 2.1. Detection of the Allopurinol Minimum Inhibitory Concentration (MIC) against P. aeruginosa PAO1

The lowest concentration of allopurinol that inhibited *P. aeruginosa* PAO1 growth (MIC) was 2 mg/mL. The subinhibitory concentration (sub-MIC or 1/10 MIC) of 200 µg/mL was used to evaluate the allopurinol anti-virulence and anti-QS activities in all experiments.

### 2.2. The Allopurinol Inhibitory Effect on P. aeruginosa PAO1 Growth at sub-MIC

**Figure 1:**
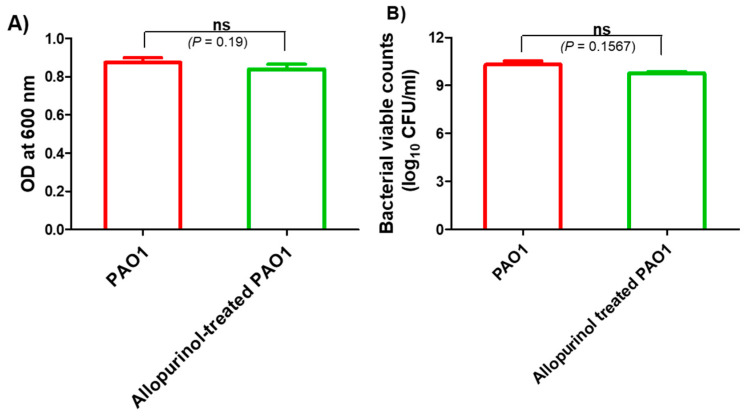
Allopurinol effect on *P. aeruginosa* PAO1 growth: (**A**) the turbidities of overnight bacterial cultures in the absence or presence of 1/10 MIC of allopurinol were measured at OD 600 nm; (**B**) viable counting of allopurinol-treated and control PAO1 cultures after overnight incubation. Allopurinol showed no statistically significant inhibitory effect on bacterial growth.

The possible inhibition of virulence and QS by allopurinol may be owed to its effect on *P. aeruginosa* PAO1 growth. To exclude such a probability, the turbidities of *P. aeruginosa* PAO1 overnight cultures provided with or without allopurinol (1/10 MIC) were measured at 600 nm. There was no significant difference between turbidities of *P. aeruginosa* overnight cultures grown in the presence or absence of allopurinol at sub-MIC. Moreover, viable counting of overnight cultures of allopurinol-treated and untreated bacteria was performed, and no significant difference was found. These results indicate that allopurinol has no effect on bacterial growth at sub-MIC, and all the tested anti-virulence and anti-QS activities of allopurinol were not a result of its inhibition of growth (Figure 1).

### 2.3. Allopurinol Diminished the Violacein Production

**Figure 2:**
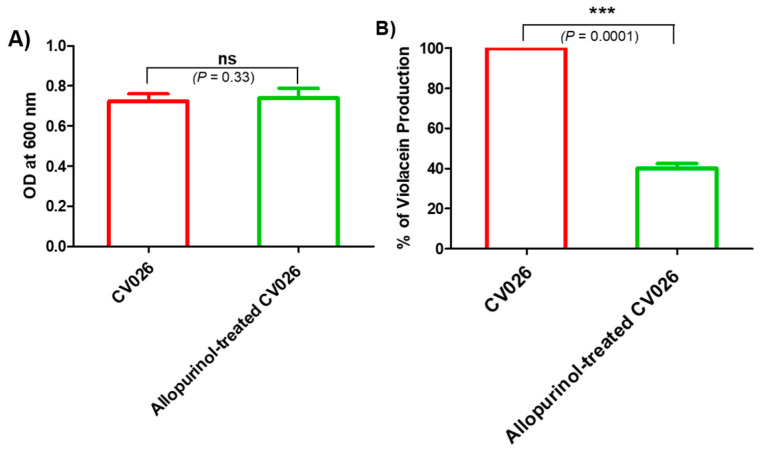
Effect of allopurinol on (**A**) *C. violaceum* CV026 growth. The turbidities of overnight bacterial cultures in the absence or presence of 1/10 MIC of allopurinol were measured at OD 600 nm. Allopurinol had no significant effect on bacterial growth. (**B**) Violacein pigment production: violacein was extracted by DMSO from the bacterial cells treated with or without 1/10 MIC of allopurinol. Allopurinol significantly reduced violacein production.

The allopurinol anti-QS activity was preliminarily assessed by measuring the decrease in the release of the QS-controlled intracellular pigment violacein by the reporter strain *C. violaceum* CV026. The biosensor, *C. violaceum* CV026, is routinely employed to assess QS due to the fact of its ability to release the pigment violacein in response to acyl-homoserine lactones under the CVi/R QS system control and, according to Harrison and Soby (2020), nearly 200 papers have been published regarding its use as an indicator for QS activity in many Gram-negative bacteria [33]. First, the allopurinol at sub-MIC effect on *C. violaceum* CV026 growth was assessed. There was no significant effect of allopurinol (sub-MIC) on *C. violaceum* CV026 growth (Figure 2A). Then, the allopurinol effect at sub-MIC on violacein production was evaluated. Significantly, allopurinol at 1/10 MIC decreased the production of violacein in *C. violaceum* CV026 by 60% (Figure 2B).

### 2.4. Allopurinol Anti-Biofilm Activity

**Figure 3:**
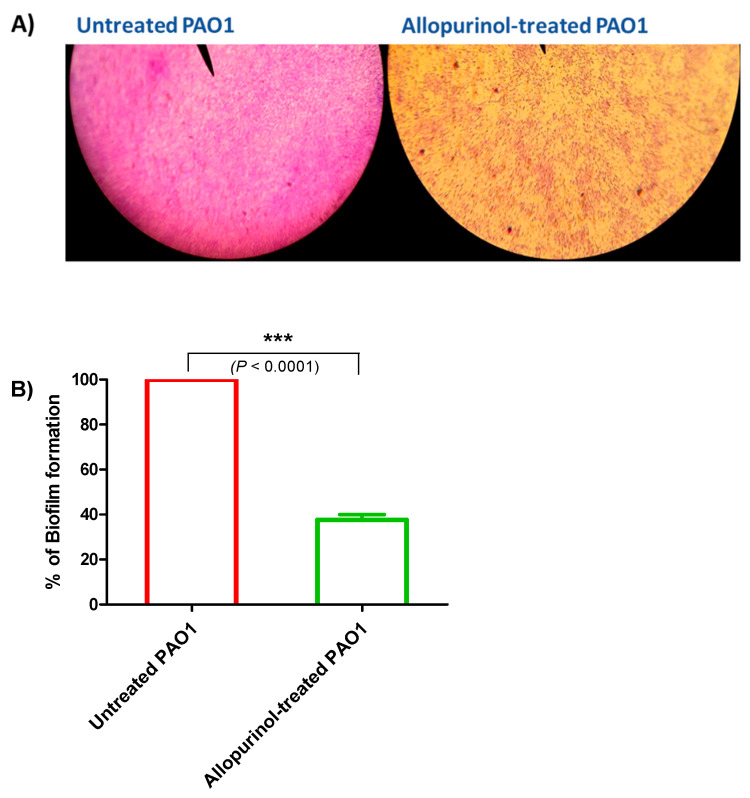
Inhibitory effect of allopurinol against biofilm formation in *P. aeruginosa* PAO1. A crystal violet assay was used to stain the biofilm-forming cells in the presence and absence of allopurinol at sub-MIC. (**A**) Light microscopic images showing a few scattered adhered *P. aeruginosa* cells when treated with allopurinol at sub-MIC. (**B**) The absorbances of the extracted crystal violet that stained the biofilm-forming cells in the presence or absence of allopurinol at sub-MIC were measured at 590 nm. Allopurinol significantly reduced biofilm formation. The data are presented as the percentage change from the untreated *P. aeruginosa* control.

Biofilm formation contributes to chronic bacterial infections; thus, its inhibition is essential for *P. aeruginosa* treatment. A crystal violet assay was used to stain the adhered bacterial biofilm-forming cells in the presence and absence of allopurinol at sub-MIC. Light microscope images were captured for the formed biofilms in the presence or absence of allopurinol at sub-MIC. The allopurinol-treated samples showed much fewer scattered adhered cells (Figure 3A). For further confirmation of the anti-biofilm effect of allopurinol at sub-MIC; the absorbances of the extracted crystal violet dye of the adhered biofilm-forming cells were measured at 590 nm in the presence or absence of allopurinol. Allopurinol exerted a significant inhibition to the formation of biofilm by *P. aeruginosa* PAO1 (61%) (Figure 3B). The data were expressed as the mean ± standard error of percentage change from the untreated *P. aeruginosa* control.

### 2.5. Inhibition of P. aeruginosa Motilities

**Figure 4:**
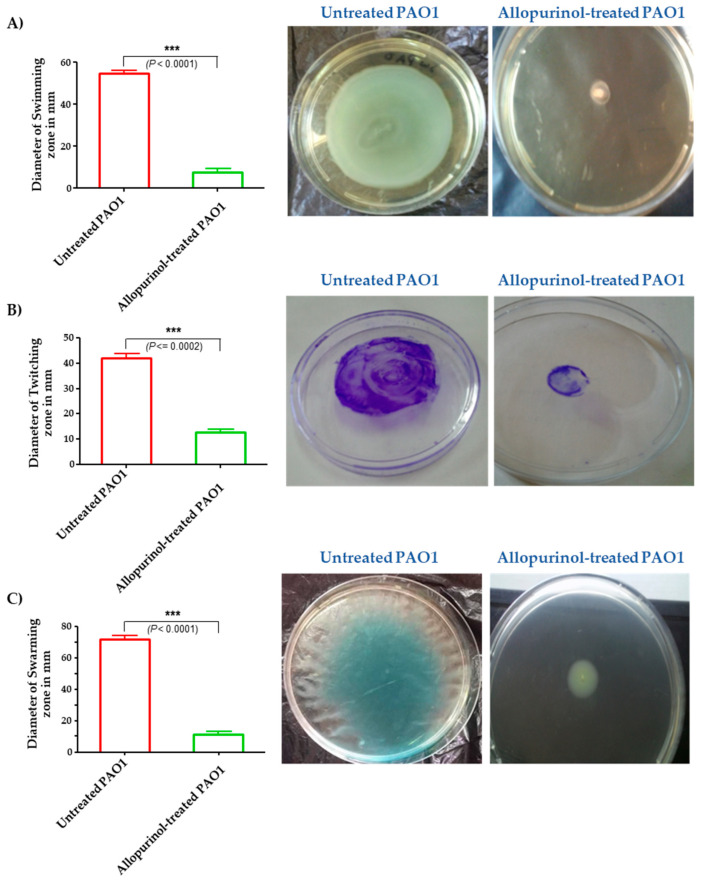
Diminishment of the (**A**) swimming, (**B**) twitching, and (**C**) swarming motilities of *P. aeruginosa* PAO1 by allopurinol. Significantly, the bacterial motilities were diminished by allopurinol at sub-MIC.

Allopurinol at sub-MIC significantly decreased *P. aeruginosa*’s ability to swim, twitch, and swarm by 92%, 87%, and 85%, respectively (Figure 4).

### 2.6. Allopurinol Diminished P. aeruginosa PAO1 QS-Controlled Virulence Factors

**Figure 5:**
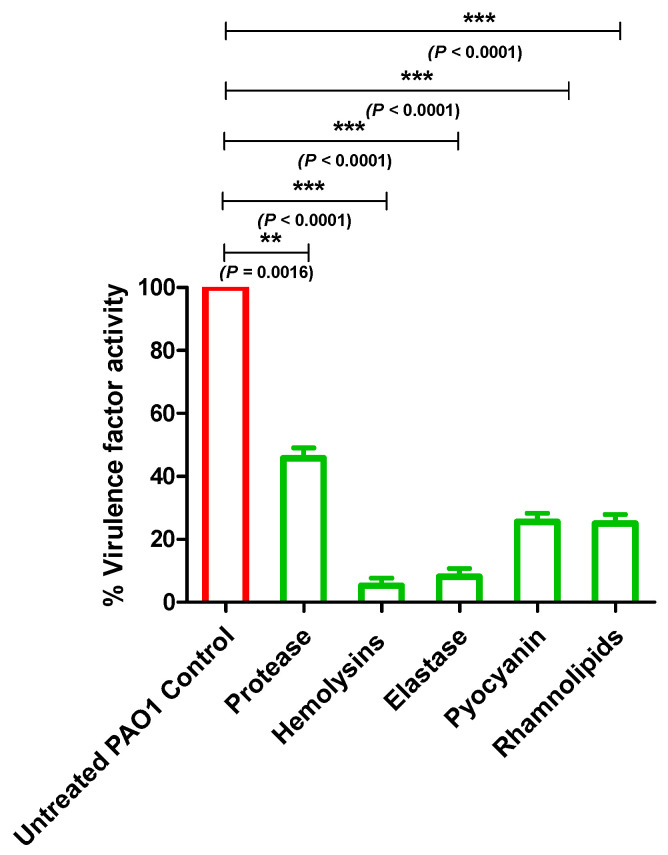
Allopurinol inhibitory effect on *P. aeruginosa* virulence. Allopurinol at sub-MIC significantly reduced the production of protease, hemolysins and elastase enzymes, pyocyanin pigment, and rhamnolipids. The supernatants of the treated and untreated allopurinol bacteria were used for the assays. Protease activity was assayed by measuring the clear zones around the wells made in skim milk agar plates. Hemolysin activity was assayed spectrophotometrically by measuring hemoglobin absorbance. Elastase activity was measured by the elastin congo red method. Pyocyanin was measured spectrophotometrically at 691 nm, and rhamnolipid was assayed using the oil displacement method by measuring the clearance zone produced by the addition of the supernatant cultures to oil. The data are presented as the percentage change from the untreated *P. aeruginosa* control.

*P. aeruginosa* QS controls the production of various extracellular enzymes and metabolites prior to their use in its host invasion. In this section, we evaluated the inhibitory effect of allopurinol at sub-MIC on the production of protease, hemolysin and elastase enzymes, pyocyanin, and rhamnolipids (Figure 5). The data are presented as the mean ± standard error of percentage change from the untreated *P. aeruginosa* control.

In order to evaluate the inhibitory activity of allopurinol at sub-MIC on the proteolytic activity of *P. aeruginosa*, a skim milk agar method was employed. The clearly formed zone around the wells in the skim milk agar, filled with collected supernatants of the allopurinol (sub-MIC) treated or untreated PAO1, were measured in mm. Allopurinol significantly reduced protease production by 55%. Moreover, the hemolysin activities of *P. aeruginosa* treated or untreated with allopurinol at sub-MIC were quantified spectrophotometrically. Allopurinol significantly inhibited the *P. aeruginosa* hemolytic activity by 95%. Elastase and protease play a key role in the pathogenesis of fatal infections caused by *P. aeruginosa* [6]. The elastin congo red method was used to assay the produced elastase enzyme by *P. aeruginosa* treated or not with allopurinol at sub-MIC. Allopurinol significantly lowered the *P. aeruginosa* elastolytic activity by 93%.

The *P. aeruginosa* arsenal of virulence factors is not restricted to only enzymes. The QS-controlled cytotoxic blue-green pigment pyocyanin is a characteristic redox metabolite that enhances *P. aeruginosa*’s cytotoxicity on the lung tissues [5]. In the presence of allopurinol at sub-MIC, pyocyanin production was significantly decreased by 74%. Rhamnolipids are glycolipid biosurfactants produced by *P. aeruginosa*. Rhamnolipid have several functions including modification of surface properties, solubilization, stimulation of motility, and biofilm formation [34]. To determine the effect of allopurinol on rhamnolipid production, the oil displacement method was performed. The clearance zone produced by the addition of the supernatants of both treated and untreated *P. aeruginosa* cultures to oil was measured. Allopurinol decreased the clearance zone by 74%.

### 2.7. Allopurinol Alleviated the Histopathological Changes in Liver and Renal Tissues in P. aeruginosa PAO1 Infected Mice

**Figure 6:**
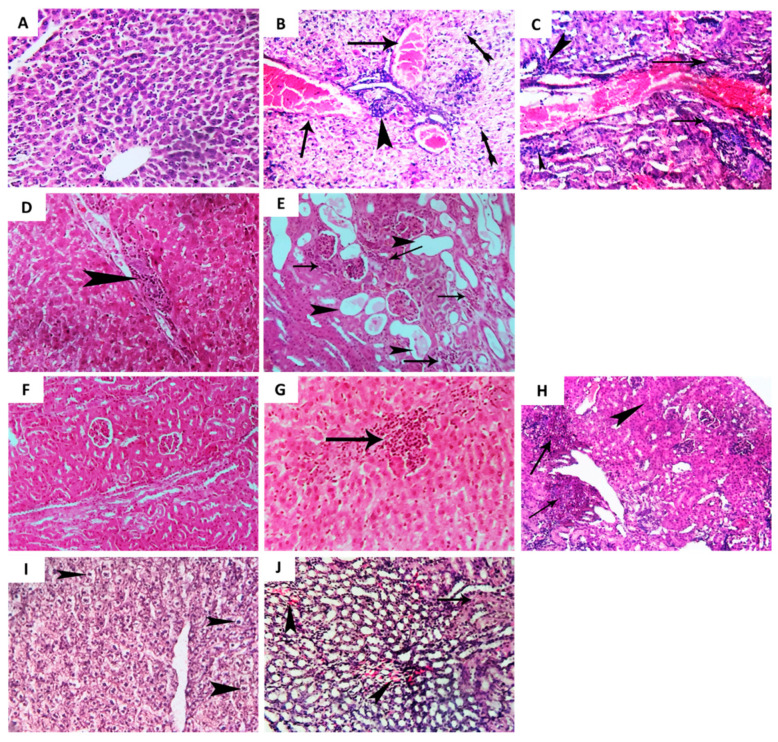
Histopathological examination of liver and renal tissues. Five groups of three-week-old albino mice (*Mus musculus*), with each group including five mice, were used in the experiment. Two negative control groups were intraperitoneally injected with sterile PBS or not infected. Two positive control groups were intraperitoneally injected with *P. aeruginosa* PAO1 (1 × 10^6^ CFU/mL) or with dimethyl sulfoxide-treated *P. aeruginosa* PAO1. The test group was injected with allopurinol-treated *P. aeruginosa* PAO1 (1 × 10^6^ CFU/mL). The mice were observed for five days and then euthanized by cervical dislocation. Both kidneys and liver were dissected from mice, rinsed with normal saline, and fixed in neutral buffered formalin (10%) for histopathological examination. Representative photomicrographs (H&E × 200) are depicted from control (un-infected), *P. aeruginosa* infected, and *P. aeruginosa* treated with allopurinol (1/10 MIC) mice groups. (**A**) Photomicrograph of mice liver from the non-infected (control) group showing apparently normal tissue architecture and cellular details. (**B**,**C**) Photomicrographs of mice liver from the *P. aeruginosa* infected group showing diffuse severe congestion of hepatic blood vessels (arrows) with perivascular cellular infiltration and colonization of *P. aeruginosa* rods (arrowheads), coagulative necrosis represented by pyknosis (tailed arrows), and the focal area of leucocytic cell infiltrated hepatic parenchyma with colonization of *P. aeruginosa* rods (arrows). (**D**,**E**) Photomicrographs of mice liver from the allopurinol-treated *P. aeruginosa* group showing mild perivascular leucocytic cells infiltration (arrowheads) with a few scattered *P. aeruginosa* rods in addition to mild congestion. (**F**) Photomicrograph of mice kidney from the non-infected (control) group showing apparently normal renal cortex with normal glomeruli and renal tubules. (**G**,**H**) Photomicrographs of mice kidney from the *P. aeruginosa* infected group showing perivascular colonization of *P. aeruginosa* (arrows) with cellular infiltration (arrowhead, (**G**)), leucocytic cell infiltration, hyperplasia of renal epithelium (arrowhead, (**H**)), and degenerated renal tubules. (**I**,**J**) Photomicrographs of mice kidney from the allopurinol-treated *P. aeruginosa* group showing mild focal infiltration of a few *P. aeruginosa* rods and less leucocytic cell infiltration (arrows (**I**)) beside milder cystic dilation of some renal tubules (arrowheads), and focal mild degeneration of some renal tubules (arrows (**J**)).

Representative photomicrographs were used to depict the effect of allopurinol at sub-MIC on relieving *P. aeruginosa*-induced pathogenesis in liver and renal tissues of infected mice. The control (un-infected) mice group showed a normal histological picture of liver and renal tissues (Figure 6A,F). The *P. aeruginosa* infected group of mice showed diffuse, severe congestion of hepatic blood vessels and coagulative necrosis associated with perivascular cellular infiltration, leucocytic cell infiltration, and colonization of *P. aeruginosa* (Figure 6B,C). Moreover, photomicrographs from renal tissues of the *P. aeruginosa* infected group of mice showed perivascular colonization of *P. aeruginosa*, leucocytic cell infiltration, hyperplasia of renal epithelium, and degenerated renal tubules (Figure 6G,H). On the other hand, allopurinol obviously diminished the colonization of *P. aeruginosa*, decreased leucocytic cell infiltration, and alleviated congestion and inflammation of the liver (Figure 6D,E) and renal (Figure 6I,J) tissues.

### 2.8. Downregulating Effect of Allopurinol on the QS-encoding Genes’ Expressions

**Figure 7:**
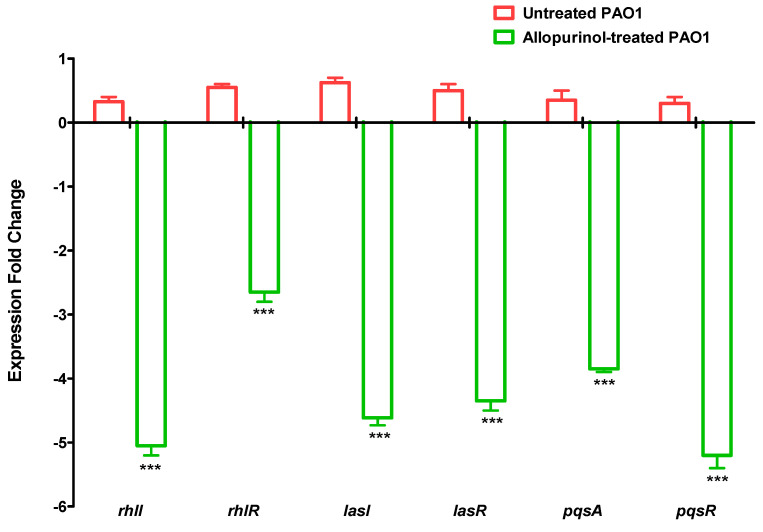
Allopurinol reduced the expressions of QS-encoding genes in *P. aeruginosa*. Allopurinol significantly reduced the expression of the genes that encode the three main QS systems in *P. aeruginosa,* namely, *rhlI*, *rhlR*, *lasI*, *lasR*, *pqsA*, and *pqsR*. *** Statistically significant difference.

The expressions of QS-encoding genes were quantified in *P. aeruginosa* treated and untreated with allopurinol at sub-MIC using qRT-PCR (Figure 7). Our findings showed a significant decrease in the expression of all the tested QS-encoding genes in *P. aeruginosa* treated with allopurinol at sub-MIC compared to untreated *P. aeruginosa*.

### 2.9. Allopurinol Competed with QS Natural Ligands in Silico

**Figure 8:**
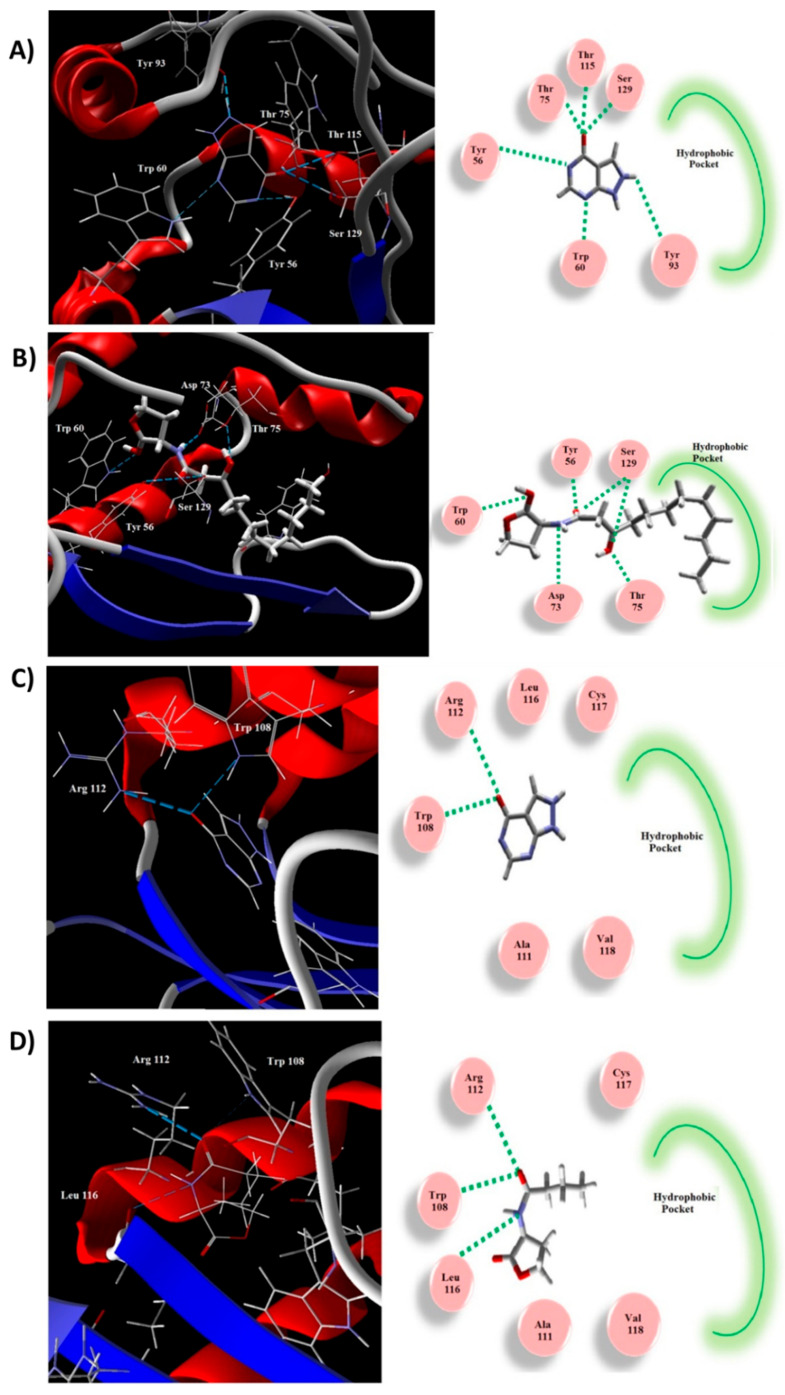
In silico allopurinol binding to QS receptors. (**A**,**C**) The molecular docking of allopurinol into the LasR and RhlR receptors’ active sites, respectively; 3D (Left) and 2D schematic views of the binding (Right). (**B**,**D**) The molecular docking of natural ligand into the active site of LasR and RhlR receptors, respectively; 3D (Left) and 2D schematic views of the binding (Right). Allopurinol binds efficiently to QS receptors and clearly exerts antagonist activity.

A molecular docking study was performed to explore the binding mode of allopurinol to the QS proteins LasR and RhlR (Figure 8). The natural ligand and allopurinol were docked into the active site of LasR. The natural ligand created H-bonds with Ser129, Asp73, Trp60, and Tyr56 (Figure 8B). The binding interaction between allopurinol and the receptor is attributed to the H-bonding with Ser129, Thr115, Tyr93, Thr75, Trp60, and Tyr56 (Figure 8A). The MolDock scores for allopurinol was −82.96 while that of the natural ligand was −164.49. The hydrophobic side-chain that persuades the proper construction of the LasR hydrophobic core was lacking in the inhibitor. The natural ligand of LasR stabilizes the conformational changes via its long acyl hydrophobic chain. Thus, allopurinol exerts LasR antagonist activity.

Similarly, allopurinol was docked into the RhlR receptor active site (Figure 8C). It revealed similar interactions to the auto-inducer C4-HSL (Figure 8D). Both created H-bonds with Arg112 and Trp108. Furthermore, C4-HSL showed an additional H-bond with the Leu112 carbonyl backbone. The binding energies of these interactions were −68.31 and −85.24 for allopurinol and C4-HSL, respectively. The C4-HSL hydrophobic acyl group is essential for the conformational changes to act as an inducer, which is not present in allopurinol, indicating it as a possible inhibitor.

## 3. Discussion

The quorum-sensing (QS) system plays the most crucial roles in orchestrating the expression of virulence factors associated with numerous pathogenic phenotypes such as bacterial adherence and biofilm formation [13,22,25,35]. Inhibition of QS is an attractive approach and is considered a novel solution to the problem of antibiotic resistance development [24,26]. Interference with QS is mostly achieved by targeting the binding of signaling molecules to the receptors by competitive inhibition. Unfortunately, many investigated QS inhibitors have not found clinical application due to the fact of their high toxicity [36,37]. In this context and because of their safety, many studies have screened various FDA-approved drugs as QS inhibitors. This hypothesis has been proven in our previous studies [6,19,20,23,25,26].

*P. aeruginosa* is a common nosocomial pathogen that causes different types of infections. One of the dangerous infections caused by *P. aeruginosa* is eye infections that can lead to blindness if untreated properly [5,29]. In this work, the hypouricemic drug allopurinol was inspected for its anti-QS activities. The allopurinol MIC was found to be 2 mg/mL, and it was used at sub-MIC (1/10 MIC or 0.2 mg/mL) to explore its QS and virulence inhibition. At sub-MIC concentration, there was no significant effect on bacterial growth and, thus, any potential allopurinol anti-QS activity cannot be attributed to its effect on the growth. In a previous study, allopurinol eye drops were applied to treat corneal burns [29]. Interestingly, allopurinol was found to inhibit QS and virulence of *P. aeruginosa* at 0.2 mg/mL in this study, a much lower concentration than that used for eye drops (4 mg/mL) [29]. This finding means that allopurinol can be used topically as eye drops to control *P. aeruginosa* eye infections.

The biosensor *C. violaceum* CV026 is routinely employed to assess QS due to the fact of its ability to release the pigment violacein in response to acyl-homoserine lactones under the CVi/R QS system control [6,36]. Preliminary investigation showed that the allopurinol at sub-MIC significantly reduced violacein production. Furthermore, a molecular in silico study was conducted to examine allopurinol’s ability to competitively occupy the QS receptors RhlR and LasR. Allopurinol could bind with both QS receptors through hydrophobic interactions and hydrogen bonds. The allopurinol docking scores, as compared to those of the natural ligands dodecanoyl homoserine lactones and butanoyl homoserine lactones, indicate the vastly promising proposed anti-QS activity of allopurinol.

Based on these primary results, further evaluations of QS-regulated virulence factors of *P. aeruginosa* in the presence of allopurinol were accomplished. QS is a main controller of biofilm formation [6,21,38], and our findings showed that allopurinol could significantly inhibit biofilm formation activity. Adhesion and biofilm formation are influenced by bacterial motility, which is regulated by QS RhlI/R and LasI/R [39,40,41]. Moreover, the strains that are QS-deficient and are diminished in motilities form thin and scattered biofilms [42,43]. In our study, allopurinol significantly inhibited the motilities of *P. aeruginosa*. Rhamnolipids are a class of rhamnose bio-surfactants, and their production is QS controlled [15,16]. Interestingly, it was shown that the impaired production of rhamnolipids in the ∆*rhlA Burkholderia glumae* mutant was responsible for defective swarming motility and can be complemented by adding exogenous rhamnolipids [44]. In this study, allopurinol significantly impaired the production of rhamnolipids, which influenced the bacterial motility and led to diminishment of the bacterial adhesion and formation of biofilms.

*P. aeruginosa* is armed by an armory rich in a wide range of virulence factors that are regulated by QS [16]. Pyocyanin is a main *P. aeruginosa* virulence factor that is involved in the creation of reactive oxygen radicals through oxidation of the cellular glutathiones concurrently accompanied with the reduction of oxygen [45,46]. Allopurinol showed a remarkable ability to decrease pyocyanin production. *P. aeruginosa* extracellularly produces various hydrolytic enzymes that facilitate bacterial spread inside host tissues and augments bacterial resistance to host defense [5,36,40,47]. The cleaving hydrolytic activity of protease against numerous essential proteins and immunoglobulins and the cytolytic activity of hemolysins play essential roles in establishing *P. aeruginosa* infection [40]. An additional QS-regulated extracellular enzyme elastase is of additional special concern, as it is believed to be the major virulence factor that causes extensive tissue damage during *P. aeruginosa* infection and facilitates its spread [48,49]. In our study, the production of elastase, hemolysin, and protease was significantly lowered by allopurinol. To evaluate allopurinol’s effect on *P. aeruginosa* pathogenesis, we injected mice with *P. aeruginosa* treated with allopurinol. Histopathological examination of liver and renal tissues showed that allopurinol diminished the *P. aeruginosa* colonization and attested to its ability to alleviate *P. aeruginosa*-induced inflammation and tissue damage. Based on the above findings that showed the considerable ability of allopurinol at sub-MIC to mitigate the QS-controlled *P. aeruginosa* virulence, it is supposed that allopurinol activity is due to its anti-QS activity.

*P. aeruginosa* virulence is regulated by three QS systems, two of which are LuxI/LuxR types, namely, LasI/LasR and RhlI/RhlR systems, and one of which is of the non-LuxI/LuxR type Pseudomonas quinolone signal (PQS) system [36,50]. Meanwhile LuxR receptors LasR and RhlR sense C12-homoserine lactone and butanoyl homoserine lactone auto-inducers, respectively; there is another orphan LuxR homolog, QscR, that binds to LasI auto-inducers [50,51]. In addition, the non-LuxR receptor, PqsR (also named MvfR), senses its own auto-inducers PqsA and others [52]. To assess the allopurinol anti-QS activity; allopurinol was in silico docked into QS receptors LasR and RhlR. The molecular docking study provided additional virtual proof of the capability of allopurinol to bind competitively to RhlR and LasR receptors and inhibit the natural auto-inducers butanoyl homoserine lactone and dodecanoyl homoserine lactone, respectively. Because of the allopurinol thiazole moiety, it can also competitively inhibit the binding of the signaling molecule 2-(2-hydroxyphenyl)-thiazole-4-carbaldehyde to QS systems. Further, the expression of *P. aeruginosa* QS receptors (LasR, RhlR, and PqsR) and their auto-inducers (LasI, RhlI, and PqsA) encoding genes were quantified. Interestingly, allopurinol significantly downregulated the expression of QS-encoding genes. These findings provide the basis for the elucidation of the observed reduction in the QS-regulated virulence factor production. The significant down-expression of the main QS system (lasI/lasR) which, as a result, may downregulate other QS systems (PQS and RhlR/I), may have resulted in a final outcome of significant allopurinol anti-virulence and anti-QS capacity.

## 4. Materials and Methods

### 4.1. Media, Chemicals, and Bacterial Strains

The used media, Luria–Bertani (LB) broth and agar, Mueller Hinton (MH) broth and agar, tryptone, and tryptone soya broth (TSB) were purchased from Oxoid (Hampshire, UK). All used chemicals were of pharmaceutical grade. The chemicals dimethyl sulphoxide (DMSO), glacial acetic acid, allopurinol, *N*-hexanoyl homoserine, elastin congo red, crystal violet, and lactone were purchased from Sigma–Aldrich (St. Louis, MO, USA). The bacterial *P. aeruginosa* PAO1 strain was obtained from the Department of Microbiology, Faculty of Pharmacy, Mansoura University, and the *C. violaceum* CV026 mutant strain was obtained from the Department of Microbiology, Faculty of Pharmacy, Ain Shams University.

### 4.2. Determination of MIC of Allopurinol

The MIC of allopurinol against *P. aeruginosa* PAO1 and *C. violaceum* CV026 were determined by the broth microdilution method according to the Clinical Laboratory and Standards Institute Guidelines (CLSI, 2015) [6,26]. Several dilutions of allopurinol (32, 16, 8, 4, 2, 1, 0.5, and 0.25 mg/mL) were prepared in Mueller–Hinton broth by two-fold serial dilution. Aliquots of 100 µl of allopurinol dilutions were delivered into the wells of microtiter plates. Then, aliquots of 100 µl of bacterial suspension in MH broth with an approximate cell density of 1 × 10^6^ CFU/mL were added to the wells. After overnight incubation of the microtiter plate at 37 °C, the growth was observed in the wells and the MIC was detected as the least concentration of allopurinol that inhibited observable growth.

### 4.3. Effect of Allopurinol at sub-MIC on the Growth of Bacteria

To ensure that allopurinol had no influence on the *P. aeruginosa* PAO1 or *C. violaceum* CV026 growth, the effect of 1/10 MIC of allopurinol on the growth of each of PAO1 and CV026 strains was determined [5,19]. Overnight culture of each bacterial strain was inoculated in LB broth containing allopurinol at sub-MIC and control LB broth without allopurinol. Then, cultured overnight at 37 °C, the bacterial turbidities were measured at 600 nm using a Biotek Spectrofluorometer (Biotek, Winooski, VT, USA). To further confirm that allopurinol had no effect on growth of PAO1, the viable counts (CFU/ML) were determined following overnight incubation at 37 °C in the presence and absence of the sub-MIC of allopurinol.

### 4.4. Violacein Inhibition Assay

As described earlier [6], *C. violaceum* CV026 was cultured overnight in LB broth and the turbidity was adjusted to 1 at 600 nm. One hundred microliters of bacterial suspensions were mixed with 100 μL aliquots provided with the auto-inducer *N*-hexanoyl-homoserine lactone in the presence and absence of allopurinol (1/10 MIC) in the wells of a 96-well microtiter plate. Then, after incubation overnight at 25 °C, it was completely dried by leaving it at 60 °C. DMSO (100 μL) was added, and the plates were incubated at 30 °C with shaking to elute violacein pigment, and a DMSO negative control was prepared. Violacein was quantified by measuring the absorbance at 590 nm using a Biotek Spectrofluorometer (Biotek, Winooski, VT, USA).

### 4.5. Biofilm Inhibition Assay

The allopurinol’s ability to inhibit biofilm formation was assessed as described before [13,19]. A bacterial suspension of PAO1 with a cell density of 1 × 10^6^ CFU/mL was prepared in TSB from fresh overnight cultures. PAO1 suspension was added in aliquots of 0.1 mL to the wells of sterile microtiter plates in the absence or presence of allopurinol at sub-MIC. After overnight incubation at 37 °C, the planktonic cells were removed, and the wells were washed. The attached cells were fixed by 99% methanol for 25 min. Crystal violet (1%) was used to stain the wells for 20 min and any excess stain was washed off before air-drying and elution of the attached dye with glacial acetic acid (33%). The absorbance of eluted dye was measured at 590 nm using the Biotek Spectrofluorometer (Biotek, Winooski, VT, USA).

### 4.6. Microscopic Visualization of Biofilm Inhibition

As described before [19], biofilms formed on the sterilized cover slips placed in 50 mL sterile Falcon tubes with TSB containing 1/10 MIC of allopurinol and control and inoculated with PAO1 suspension (OD of 1 at 600 nm). After 16 h incubation at 37 °C, the non-adherent cells were removed from the cover slips by washing. The biofilm-forming cells were methanol-fixed, crystal violet stained, and inspected under a high power light microscope (400× magnification) using a Leica DM750 HD digital microscope (Mannheim, Germany).

### 4.7. Swimming, Twitching, and Swarming Motilities Assay

The possible effect of allopurinol on inhibiting swarming, swimming, and twitching motilities was performed as described earlier [5,6]. Swimming agar plates (0.3% agar) with allopurinol (1/10 MIC) and allopurinol-free control plates were center stabbed with 5 µL of diluted overnight tryptone broth cultures of PAO1. The swimming zones were measured after overnight incubation of the plates at 37 °C. Considering the evaluation of twitching motility inhibition, 1% LB agar plates with and without allopurinol (1/10 MIC) were stab-inoculated with 2 µL of the overnight tryptone broth cultures of PAO1. After incubation at 37 °C for 48 h, the agar was removed and the plates were air-dried and crystal violet stained. Then, after removal of the stain, the plates were washed and dried, and the zones of twitching were measured. The swarming motility inhibition was assessed by surface inoculation of allopurinol-containing agar plates and allopurinol-free agar plates (0.5% agar) with 2 µL of diluted overnight *P. aeruginosa* PAO1 cultures. The swarming zones were measured after incubation at 37 °C for 16 h.

### 4.8. Protease Inhibition Assay

The proteolytic inhibition activity of allopurinol was investigated by the skim milk agar method [19,20]. LB broth tubes provided with or without 1/10 MIC of allopurinol were cultured with *P. aeruginosa* PAO1 overnight at 37 °C. After centrifugation to obtain supernatants, the supernatants (100 µL) were added to the wells made in skim milk agar plates (5%). After overnight incubation at 37 °C, the clear zones due to the proteolytic activity were measured.

### 4.9. Elastase Inhibition Assay

The elastin-congo red assay was used to estimate the anti-elastolytic activity of allopurinol [6]. First, elastin-congo red reagent was prepared: 10 mg of elastin-congo red in 500 µL buffer (0.1 mol/L Tris pH 7.2 and 10 mol/L CaCl_2_). Supernatants of allopurinol treated and untreated PAO1 cultures were collected. Aliquots of 0.5 mL were mixed with the elastin-congo red reagent. The reaction tubes were incubated with shaking at 37 °C for 6 h. Then, they were centrifuged to remove the insoluble elastin congo red, and finally the absorbance was measured at 495 nm using a Biotek Spectrofluorometer (Biotek, Winooski, VT, USA).

### 4.10. Pyocyanin Inhibition Assay

The allopurinol’s ability to reduce the production of the pigment pyocyanin by *P. aeruginosa* was evaluated [6,45]. In LB broth, *P. aeruginosa* PAO1 was cultured, incubated overnight, and the bacterial suspension was adjusted to an OD of 0.4 at 600 nm. Ten microliters of the prepared bacterial suspensions were added to LB broth tubes (1 mL) containing 1/10 MIC of allopurinol and to the control tubes. Then, they were incubated at 37 °C for 48 h. To separate the supernatants, the tubes were centrifuged, and the pyocyanin inhibition was assessed by measuring the absorbance at 691 nm using a Biotek Spectrofluorometer (Biotek, Winooski, VT, USA).

### 4.11. Hemolysin Inhibition Assay

The hemolysin activity of *P. aeruginosa* in the presence and absence of allopurinol was estimated [19,47]. The prepared supernatant (0.5 mL) was added to 0.7 mL of a fresh erythrocyte suspension in 2% saline and incubated at 37 °C for 2 h. The absorbance of released hemoglobin from lysed erythrocytes was measured at 540 nm (using a Biotek Spectrofluorometer; Biotek, Winooski, VT, USA) in the supernatants separated by centrifugation at 4 °C. The released hemoglobin was compared with a prepared positive control (0.1% SDS in erythrocyte suspension) and negative control (erythrocytes in LB broth). The percentage of hemolysis was calculated: percentage hemolysis = (X-B/T-B) × 100, (X: the absorbance in the case of treated or untreated samples; B: the absorbance in the case of the negative control; T: the absorbance in the case of the positive control). The hemolysis in cultures treated with allopurinol was measured as a % compared to hemolysis in allopurinol untreated culture.

### 4.12. Rhamnolipids Assay

The assay of rhamnolipid production was performed using the oil displacement method [34,53]. Cell-free supernatants of allopurinol-treated and untreated *P. aeruginosa* PAO1 were prepared. Crude oil (20 μL) (purchased from Misr Petroleum Company, Egypt) was mixed with 15 mL of distilled water in a Petri dish, and then 10 μL of supernatant was inoculated to the oil drop. The displacement and clearing zone were observed, and the clearing zone diameter, which is related to rhamnolipid surfactant activity, was measured.

### 4.13. Quantitative RT-PCR of QS Genes

To confirm the inhibitory activity of allopurinol against QS and virulence of *P. aeruginosa* PAO1, quantitative real-time PCR was used [5,6]. First, the RNA of both allopurinol treated and untreated *P. aeruginosa* PAO1 cultures was extracted by the Gene JET RNA Purification Kit (Thermoscientific, 168 Third Avenue Waltham, MA, USA) according to the instructions of the manufacturer, and the obtained RNA was stored at −70 °C until use. The levels of relative expression of QS-encoding genes were evaluated in *P. aeruginosa* PAO1 strain treated and untreated with allopurinol at sub-MIC by qRT-PCR. The housekeeping *ropD* gene was employed to normalize the relative expression levels of tested genes. The primers used in this study was listed earlier [6], and the protocol of SensiFAST™ SYBR^®^ Hi-ROX One-Step Kit (Bioline, UK) was used for analysis using the. StepOne Real-Time PCR system (Applied Biosystem, San Franscisco, CA, USA). To confirm the specific PCR amplification, agarose gel electrophoresis and a melting curve analysis of products were used according to the manufacturer’s recommendation. The relative gene expression was calculated by the comparative threshold cycle (∆∆Ct) method [5,6,54].

### 4.14. In Silico Molecular Docking Study

An silico study was conducted to detect the molecular interaction of allopurinol with the LasR and RhlR QS receptors of *P. aeruginosa* PAO1. The RhlR receptor model (ID: P54292.1) was obtained from the protein model portal, and the crystal structure of LasR ligand binding domain was found in the Protein Data Bank (PDB ID: 2 UV0) [5,36]. Both allopurinol and the natural ligand, 3-oxo-dodecanoyl homoserine lactone or butanoyl homoserine lactone (C4-HSL), were docked into the active site of the LasR or RhlR receptors, respectively, as described earlier [5]. The most energetically favored conformer was saved for docking, and the top returned poses were chosen for analysis. The AutoDockTools software was employed to generate the pdbqt files after determining the atomic partial charges using the Gasteiger method using the suitable setting that were used for autodock via docking into the LasR and rhlR active sites as described earlier [5].

### 4.15. Histopathological Study

In optimal housing conditions, three-week-old albino mice (*Mus musculus*) were allowed to acclimatize before starting the experiment. The used protocol and animal handling procedures were approved by the Ethical Committee for Animal Handling at Zagazig University (ECAH ZU, 18 June 2019), Faculty of Pharmacy, Zagazig University, Egypt, based on the Weatherall report recommendations. The study embraced 5 groups of 5 mice each. Two negative control groups were intraperitoneally injected with sterile PBS or not infected. Two positive control groups were intraperitoneally injected with *P. aeruginosa* PAO1 (1 × 10^6^ CFU/mL) or with the used solvent dimethyl sulfoxide-treated *P. aeruginosa* PAO1. The test group was injected with allopurinol-treated *P. aeruginosa* PAO1 (1 × 10^6^ CFU/mL). The mice were observed for five days and then were euthanized by cervical dislocation. Both kidneys and liver were dissected from mice, rinsed with normal saline, and fixed in neutral buffered formalin (10%) for histopathological examination. After the dehydration in increasing concentrations of ethyl alcohol, the samples were cleared in xylol, impregnated, and embedded in paraffin wax. Rotatory microtome was employed to cut 5 μm thick sections, then stained with hematoxylin and eosin (H&E × 200) stain and examined using a Leica DM750 HD digital microscope (Mannheim, Germany) [46,55].

### 4.16. Statistical Analysis

All the tests were performed in triplicates and presented as the means ± standard errors. Unpaired *t*-tests via GraphPad Prism 8 (GraphPad Software, Inc., San Diego, CA, USA) were used to evaluate the significance of the allopurinol inhibitory activities against bacterial motilities. Paired *t*-tests via GraphPad Prism 8 were used to evaluate the significance of the allopurinol’s effects against growth, biofilm formation, and other virulence factors. One-way ANOVA tests followed by Dunnett post-test were used to evaluate the significance of the downregulation of the QS genes by quantitative real-time PCR. *p*-Values < 0.05 were considered statistically significant.

## 5. Conclusions

Bacterial resistance is an increasing obstacle that must be resolved. Targeting bacterial virulence may be a good approach to disarm the bacterial virulence and enable the immune system to eradicate the bacteria. In this study, we aimed to repurpose allopurinol by evaluating its anti-virulence and anti-QS activities. Interestingly, allopurinol showed a significant ability to reduce the QS-regulated virulence factors in *P. aeruginosa*. Furthermore, allopurinol obviously reduced *P. aeruginosa* colonization and diminished the associated pathological effects on the liver and renal tissues of mice. As hypothesized, the anti-virulence activity of allopurinol can be attributed to its QS-inhibiting activities in *P. aeruginosa*. Allopurinol significantly downregulated QS-encoding genes and showed considerable ability to hinder the binding of autoinducers to QS receptors in silico. This work highlights the anti-virulence and anti-QS activities of allopurinol, keeping the door open for more detailed pharmacological and pharmaceutical studies for repurposing allopurinol and related chemical moieties for the treatment of bacterial infections.

## References

[1] Willcox M.D. (2007). *Pseudomonas aeruginosa* infection and inflammation during contact lens wear: A review. Optom. Vis. Sci..

[2] Church D., Elsayed S., Reid O., Winston B., Lindsay R. (2006). Burn wound infections. Clin. Microbiol. Rev..

[3] Klockgether J., Tummler B. (2017). Recent advances in understanding *Pseudomonas aeruginosa* as a pathogen. F1000Research.

[4] Hilliam Y., Kaye S., Winstanley C. (2020). *Pseudomonas aeruginosa* and microbial keratitis. J. Med. Microbiol..

[5] Aldawsari M.F., Khafagy E.S., Saqr A.A., Alalaiwe A., Abbas H.A., Shaldam M.A., Hegazy W.A.H., Goda R.M. (2021). Tackling Virulence of *Pseudomonas aeruginosa* by the Natural Furanone Sotolon. Antibiotics.

[6] Hegazy W.A.H., Khayat M.T., Ibrahim T.S., Nassar M.S., Bakhrebah M.A., Abdulaal W.H., Alhakamy N.A., Bendary M.M. (2020). Repurposing Anti-diabetic Drugs to Cripple Quorum Sensing in *Pseudomonas aeruginosa*. Microorganisms.

[7] Valentini M., Gonzalez D., Mavridou D.A., Filloux A. (2018). Lifestyle transitions and adaptive pathogenesis of *Pseudomonas aeruginosa*. Curr. Opin. Microbiol..

[8] Stewart P.S., Costerton J.W. (2001). Antibiotic resistance of bacteria in biofilms. Lancet.

[9] Llanes C., Hocquet D., Vogne C., Benali-Baitich D., Neuwirth C., Plesiat P. (2004). Clinical strains of *Pseudomonas aeruginosa* overproducing MexAB-OprM and MexXY efflux pumps simultaneously. Antimicrob. Agents Chemother..

[10] Munita J.M., Arias C.A. (2016). Mechanisms of Antibiotic Resistance. Microbiol. Spectr..

[11] Bhargava N., Sharma P., Capalash N. (2010). Quorum sensing in Acinetobacter: An emerging pathogen. Crit. Rev. Microbiol..

[12] Askoura M., Hegazy W.A.H. (2020). Ciprofloxacin interferes with Salmonella Typhimurium intracellular survival and host virulence through repression of Salmonella pathogenicity island-2 (SPI-2) genes expression. Pathog. Dis..

[13] Hegazy W.A.H., Abbas H.A. (2017). Evaluation of the role of SsaV ‘Salmonella pathogenicity island-2 dependent type III secretion system components on the virulence behavior of Salmonella enterica serovar Typhimurium. Afr. J. Biotechnol..

[14] Smith R.S., Iglewski B.H. (2003). *Pseudomonas aeruginosa* quorum sensing as a potential antimicrobial target. J. Clin. Investig..

[15] Jimenez P.N., Koch G., Thompson J.A., Xavier K.B., Cool R.H., Quax W.J. (2012). The multiple signaling systems regulating virulence in *Pseudomonas aeruginosa*. Microbiol. Mol. Biol. Rev..

[16] Lee J., Zhang L. (2015). The hierarchy quorum sensing network in *Pseudomonas aeruginosa*. Protein Cell.

[17] Al Saqr A., Khafagy E.S., Alalaiwe A., Aldawsari M.F., Alshahrani S.M., Anwer M.K., Khan S., Lila A.S.A., Arab H.H., Hegazy W.A.H. (2021). Synthesis of Gold Nanoparticles by Using Green Machinery: Characterization and In Vitro Toxicity. Nanomaterials.

[18] Khayyat A.N., Hegazy W.A.H., Shaldam M.A., Mosbah R., Almalki A.J., Ibrahim T.S., Khayat M.T., Khafagy E.S., Soliman W.E., Abbas H.A. (2021). Xylitol Inhibits Growth and Blocks Virulence in *Serratia marcescens*. Microorganisms.

[19] Abbas H.A., Hegazy W.A.H. (2020). Repurposing anti-diabetic drug “Sitagliptin” as a novel virulence attenuating agent in *Serratia marcescens*. PLoS ONE.

[20] Abbas H.A., Hegazy W.A.H. (2017). Targeting the virulence factors of *Serratia marcescens* by ambroxol. Roum. Arch. Microbiol. Immunol..

[21] El-Hamid M.I.A., El-Naenaeey E.-S.Y., Kandeel T.M., Hegazy W.A.H., Mosbah R.A., Nassar M.S., Bakhrebah M.A., Abdulaal W.H., Alhakamy N.A., Bendary M.M. (2020). Promising Antibiofilm Agents: Recent Breakthrough against Biofilm Producing Methicillin-Resistant *Staphylococcus aureus*. Antibiotics.

[22] Bendary M.M., Ibrahim D., Mosbah R.A., Mosallam F., Hegazy W.A.H., Awad N.F.S., Alshareef W.A., Alomar S.Y., Zaitone S.A., El-Hamid M.I.A. (2020). Thymol Nanoemulsion: A New Therapeutic Option for Extensively Drug Resistant Foodborne Pathogens. Antibiotics.

[23] Vishwa B., Moin A., Gowda D.V., Rizvi S.M.D., Hegazy W.A.H., Abu Lila A.S., Khafagy E.S., Allam A.N. (2021). Pulmonary Targeting of Inhalable Moxifloxacin Microspheres for Effective Management of Tuberculosis. Pharmaceutics.

[24] Hegazy W.A.H. (2016). Diclofenac inhibits virulence of *Proteus mirabilis* isolated from diabetic foot ulcer. Afr. J. Microbiol. Res..

[25] Hegazy W.A.H., Khayat M.T., Ibrahim T.S., Youns M., Mosbah R., Soliman W.E. (2021). Repurposing of antidiabetics as *Serratia marcescens* virulence inhibitors. Braz. J. Microbiol..

[26] Khayyat A.N., Abbas H.A., Mohamed M.F.A., Asfour H.Z., Khayat M.T., Ibrahim T.S., Youns M., Khafagy E.-S., Abu Lila A.S., Safo M.K. (2021). Not Only Antimicrobial: Metronidazole Mitigates the Virulence of *Proteus mirabilis* Isolated from Macerated Diabetic Foot Ulcer. Appl. Sci..

[27] Komoriya K., Osada Y., Hasegawa M., Horiuchi H., Kondo S., Couch R.C., Griffin T.B. (1993). Hypouricemic effect of allopurinol and the novel xanthine oxidase inhibitor TEI-6720 in chimpanzees. Eur. J. Pharmacol..

[28] Osada Y., Tsuchimoto M., Fukushima H., Takahashi K., Kondo S., Hasegawa M., Komoriya K. (1993). Hypouricemic effect of the novel xanthine oxidase inhibitor, TEI-6720, in rodents. Eur. J. Pharmacol..

[29] Sekundo W., Augustin A.J., Strempel I. (2002). Topical allopurinol or corticosteroids and acetylcysteine in the early treatment of experimental corneal alkali burns: A pilot study. Eur. J. Ophthalmol..

[30] Pacher P., Nivorozhkin A., Szabo C. (2006). Therapeutic effects of xanthine oxidase inhibitors: Renaissance half a century after the discovery of allopurinol. Pharmacol. Rev..

[31] Ashihara H., Crozier A. (2001). Caffeine: A well known but little mentioned compound in plant science. Trends Plant Sci..

[32] Norizan S.N., Yin W.F., Chan K.G. (2013). Caffeine as a potential quorum sensing inhibitor. Sensors.

[33] Harrison A.M., Soby S.D. (2020). Reclassification of *Chromobacterium violaceum* ATCC 31532 and its quorum biosensor mutant CV026 to *Chromobacterium subtsugae*. AMB Express.

[34] Wozniak-Karczewska M., Myszka K., Sznajdrowska A., Szulc A., Zgola-Grzeskowiak A., Lawniczak L., Corvini P.F., Chrzanowski L. (2017). Isolation of rhamnolipids-producing cultures from faeces: Influence of interspecies communication on the yield of rhamnolipid congeners. N. Biotechnol..

[35] Askoura M., Youns M., Halim Hegazy W.A. (2020). Investigating the influence of iron on Campylobacter jejuni transcriptome in response to acid stress. Microb Pathog.

[36] Bottomley M.J., Muraglia E., Bazzo R., Carfi A. (2007). Molecular insights into quorum sensing in the human pathogen *Pseudomonas aeruginosa* from the structure of the virulence regulator LasR bound to its autoinducer. J. Biol. Chem..

[37] Aldawsari M.F., Alalaiwe A., Khafagy E.S., Al Saqr A., Alshahrani S.M., Alsulays B.B., Alshehri S., Abu Lila A.S., Danish Rizvi S.M., Hegazy W.A.H. (2021). Efficacy of SPG-ODN 1826 Nanovehicles in Inducing M1 Phenotype through TLR-9 Activation in Murine Alveolar J774A.1 Cells: Plausible Nano-Immunotherapy for Lung Carcinoma. Int. J. Mol. Sci..

[38] El-Mowafy S.A., El Galil K.H.A., El-Messery S.M., Shaaban M.I. (2014). Aspirin is an efficient inhibitor of quorum sensing, virulence and toxins in *Pseudomonas aeruginosa*. Microb. Pathog..

[39] Hema M., Vasudevan S., Balamurugan P., Adline Princy S. (2017). Modulating the Global Response Regulator, LuxO of V. cholerae Quorum Sensing System Using a Pyrazine Dicarboxylic Acid Derivative (PDCA(py)): An Antivirulence Approach. Front. Cell. Infect. Microbiol..

[40] Lyczak J.B., Cannon C.L., Pier G.B. (2000). Establishment of *Pseudomonas aeruginosa* infection: Lessons from a versatile opportunist. Microbes Infect..

[41] Winzer K., Williams P. (2001). Quorum sensing and the regulation of virulence gene expression in pathogenic bacteria. Int. J. Med. Microbiol..

[42] Lee D.J., Jo A.R., Jang M.C., Nam J., Choi H.J., Choi G.W., Sung H.Y., Bae H., Ku Y.G., Chi Y.T. (2018). Analysis of two quorum sensing-deficient isolates of *Pseudomonas aeruginosa*. Microb. Pathog..

[43] Mayer C., Muras A., Parga A., Romero M., Rumbo-Feal S., Poza M., Ramos-Vivas J., Otero A. (2020). Quorum Sensing as a Target for Controlling Surface Associated Motility and Biofilm Formation in *Acinetobacter baumannii* ATCC((R)) 17978(TM). Front. Microbiol..

[44] Nickzad A., Lepine F., Deziel E. (2015). Quorum Sensing Controls Swarming Motility of Burkholderia glumae through Regulation of Rhamnolipids. PLoS ONE.

[45] Das T., Manefield M. (2012). Pyocyanin promotes extracellular DNA release in *Pseudomonas aeruginosa*. PLoS ONE.

[46] Hegazy W.A.H., Henaway M. (2015). Hepatitis C virus pathogenesis: Serum IL-33 level indicates liver damage. Afr. J. Microbiol. Res..

[47] Rossignol G., Merieau A., Guerillon J., Veron W., Lesouhaitier O., Feuilloley M.G., Orange N. (2008). Involvement of a phospholipase C in the hemolytic activity of a clinical strain of *Pseudomonas fluorescens*. BMC Microbiol..

[48] Nomura K., Obata K., Keira T., Miyata R., Hirakawa S., Takano K., Kohno T., Sawada N., Himi T., Kojima T. (2014). *Pseudomonas aeruginosa* elastase causes transient disruption of tight junctions and downregulation of PAR-2 in human nasal epithelial cells. Respir. Res..

[49] Salunkhe P., Smart C.H., Morgan J.A., Panagea S., Walshaw M.J., Hart C.A., Geffers R., Tummler B., Winstanley C. (2005). A cystic fibrosis epidemic strain of *Pseudomonas aeruginosa* displays enhanced virulence and antimicrobial resistance. J. Bacteriol..

[50] Rutherford S.T., Bassler B.L. (2012). Bacterial quorum sensing: Its role in virulence and possibilities for its control. Cold Spring Harb. Perspect. Med..

[51] Lintz M.J., Oinuma K., Wysoczynski C.L., Greenberg E.P., Churchill M.E. (2011). Crystal structure of QscR, a *Pseudomonas aeruginosa* quorum sensing signal receptor. Proc. Natl. Acad. Sci. USA.

[52] Xiao G., He J., Rahme L.G. (2006). Mutation analysis of the *Pseudomonas aeruginosa* mvfR and pqsABCDE gene promoters demonstrates complex quorum-sensing circuitry. Microbiology.

[53] Deodhar S., Rohilla P., Manivannan M., Thampi S.P., Basavaraj M.G. (2020). Robust Method to Determine Critical Micelle Concentration via Spreading Oil Drops on Surfactant Solutions. Langmuir.

[54] Youns M., Askoura M., Abbas H.A., Attia G.H., Khayyat A.N., Goda R.M., Almalki A.J., Khafagy E.S., Hegazy W.A.H. (2021). Celastrol Modulates Multiple Signaling Pathways to Inhibit Proliferation of Pancreatic Cancer via DDIT3 and ATF3 Up-Regulation and RRM2 and MCM4 Down-Regulation. Oncol. Targets Ther..

[55] Emara N.A., Mahmoud M.F., El Fayoumi H.M., Mahmoud A.A.A. (2021). The renoprotective effect of glycyrrhizic acid in insulin-resistant rats exposed to aluminum involves the inhibition of TLR4/NF-kappaB signaling pathway. Naunyn Schmiedebergs Arch. Pharm..

